# TO TELL OR NOT TO TELL: TYPOLOGIES OF OLDER ADULTS PREFERENCES ON DIAGNOSIS DISCLOSURE OF CRITICAL ILLNESS IN CHINA

**DOI:** 10.1093/geroni/igac059.2507

**Published:** 2022-12-20

**Authors:** Yifan Lou, Jinyu Liu

**Affiliations:** Columbia University, New York, New York, United States; Columbia University, New York City, New York, United States

## Abstract

**Background:**

A priori of advance care planning, that older adults should know their diagnosis, is not guaranteed nor legally supported in China. Typically, doctors will inform the family members of the diagnosis and prognosis of critical illness and let family members decide whether to inform or not. This study aims to explore how older Chinese prefer diagnosis disclosure of their critical illness and the factors related to each typology of desired roles.

**Methods:**

We surveyed 571 older adults in Shanghai from late-2021. We included 7 items measuring values of diagnosis disclosure on three levels: to self, to significant others, and regarding physician disclosure approach. We characterized preference types using latent class analysis. Multinomial regression models on class memberships were used with cultural, sociodemographic, and healthcare experiences predictors.

**Results:**

Three latent classes were identified: 34% of respondents preferred control over own diagnosis and respected significant others’ rights to know their own diagnosis (“transparent”). 50% of respondents has conflicted values. They preferred to know own diagnosis and to discuss with physicians privately, but chose to conceal others’ diagnoses if their significant others have serious illness (“contradictory”). 16% preferred to delegate control over diagnosis to others and assumes that their families hold the same value (“avoidant”). Increased familism is significantly related to contradictory type. People with experience of hospitalization and making medical decisions for family members were more likely to be transparent. Discussions: We discussed research, practice, and policy implications on culturally-appropriate approaches to honor patients’ autonomy with serious illness in China.

